# How, Why and When: Nursing Staff's Experiences of Working With Suicide Risk Assessment Instruments

**DOI:** 10.1002/nop2.70068

**Published:** 2024-11-03

**Authors:** Rikard Wärdig, Isabella Wallerstedt, Anna Mattison Nyström, Sally Hultsjö

**Affiliations:** ^1^ Division of Nursing and Reproductive Health Department of Health Medicine and Caring Sciences Linköping University Linköping Sweden; ^2^ Department of Psychiatry Ryhov County Hospital Jönköping Sweden

**Keywords:** intuition, nurse, qualitative method, suicide prevention, suicide risk assessment instruments

## Abstract

**Aims and objectives:**

To describe psychiatric nursing staff´s experiences of working with suicide risk assessment instruments.

**Background:**

Around the world, approximately 720,000 people die by suicide each year, of which almost 20% have an ongoing contact with specialist psychiatry during their last month alive. To identify which patients have an increased risk of suicide is a highly important task for nursing staff. Suicidal behaviour is complex and unpredictable. Nursing staff work closely with patients in everyday psychiatric care and often possess unique patient knowledge. These professionals must therefore be able to know when a patient's eventual suicidality requires further attention and efforts.

**Design:**

A descriptive qualitative study.

**Methods:**

Convenience sampling including nursing staff with more than 2 years of experience in specialist psychiatry. The data collection took place through semi‐structured interviews and was then analysed according to conventional content analysis.

**Results:**

The nursing staff felt that a natural conversation is superior suicide risk assessment instruments and that experience and patient knowledge are the primary factors for facilitating a suicide risk assessment. This meant that some nursing staff rarely used suicide risk assessment instruments, although they sometimes could be a useful support in the conversation and provided a sense of personal security when documenting results.

**Conclusion:**

Suicide risk assessment instrument can be significant and helpful in certain cases, but the essential components of a suicide risk assessment consist of a conversation to understand the meaning of suicide from the patient's perspective.

**Patient or Public Contribution:**

Psychiatric nursing staff contributed to this study by sharing their experiences through interviews.

## 1 Introduction

There are approximately 720,000 suicides annually worldwide, with 20 suicide attempts for every completed suicide (WHO 2023). Of those who died by suicide, around 90% had an underlying mental illness, most commonly major depression (Olfson, Blanco, and Marcus 2016; Bachmann 2018). Individuals at risk of suicide frequently visit both primary care and specialised psychiatric care. Among those who died by suicide, approximately 45% had been in contact with healthcare professionals in primary care during the last month of their lives. Twenty per cent had been in contact with specialised psychiatric care (Ahmedani et al. 2014), the unit responsible for patients with specific, more severe psychiatric diagnoses. Thus, a significant portion of suicides could be prevented if identified.

## 2 Background

Specialised psychiatric care bears the principal responsibility for the detection and management of patients exhibiting suicidal ideation. Suicidal ideation involves thoughts or plans about suicide, ranging from a vague desire to die to detailed plans with intent to act. It is a major risk factor for future suicide attempts and deaths, often seen as a spectrum from passive thoughts to active planning and behaviour (Han et al. 2016). Some individuals may attempt suicide without prior ideation, though this is debated due to potential underreporting (Harmer et al. 2023). A useful analogy is to see suicidal ideation as the larger, unseen part of an iceberg, with the act of suicide as the visible tip. This highlights the need for early identification and intervention. Nurses, in virtue of their extended durations of interaction with patients relative to other healthcare professionals, occupy a pivotal position for the identification of individuals harbouring suicidal ideation (Berglund, Åström, and Lindgren 2016).

Although it is impossible to predict a suicidal act, certain factors warrant closer examination of the individual. Factors such as feelings of hopelessness, depression, stress and maladaptive coping strategies involving guilt and distraction can predict suicidal behaviour (Rudd et al. 2006). Additionally, patients exhibiting alexithymic personality traits are overrepresented in terms of suicidal ideation (De Berardis et al. 2020). Research has demonstrated that individuals at high risk for suicide tend to employ negative coping strategies due to a lack of knowledge about other, beneficial, coping strategies (Lew et al. 2019). Additionally, severe somatic illness (McFarland et al. 2019) and self‐harming behaviours (Carroll, Metcalfe, and Gunnel 2014) are factors that further increase the risk of suicide.

Assessing suicide risk is one of the most challenging tasks in psychiatry as ahead of the suicide, the individual undergoes two processes in parallel (Ovox et al. 2023). The individual is partly planning life and partly thinking about how to die by suicide. We must therefore not see forward‐looking behaviour as a sure sign that the patient is not suicidal. Another challenge is that suicidal ideation more often leads to action among older people, while suicide ideation in younger people had significantly fewer suicide deaths (Harmer et al. 2023). In Sweden, to identify individuals at risk of suicide, suicide risk assessments are conducted through conversations between healthcare providers and patients, combined with the use of assessment instruments that can serve as an educational support (Runeson et al. 2017).

It is important to be aware that several systematic literature reviews have been conducted to evaluate these instruments. The conclusions drawn from these reviews indicate a lack of scientific support for any assessment instrument to have sufficient reliability in predicting future suicide (SBU 2015; Runeson et al. 2017). A significant concern is that these instruments predominantly target depression and low mood. Although these conditions are frequently linked to suicide, it is important to note that the prevalence of depression is substantially higher than the incidence of suicide (the Global Burden of Disease Study 2018; Leavey et al. 2017). In a systematic scoping review conducted in the UK, it was found that most articles concerning how suicide risk is assessed in healthcare settings tended to focus on risk factors, such as depressive symptoms (Fedorowicz et al. 2023). They advocate that protective factors, which may buffer against suicide, should be considered in the assessments. Additionally, the patient's perceived social support and life satisfaction are valuable in the assessment, as these factors can reduce the association between suicidal ideation and depression (Siegmann et al. 2018). The interpersonal encounter in suicidal risk assessments is therefore of utmost importance.

Wanting to die is a process that is confusing and involves suffering for the person; confirming the suffering, and creating trust and closeness, is primary in the care of the patient (Berg, Rørtveit, and Aase 2017) and can be what turns around the suicidal process (Talseth, Gilje, and Norberg 2003). This highlights the importance of meeting a suffering person and listening to their feelings in order to understand the meaning and function of death for the specific individual. When caring for suicidal persons, healthcare professionals tend to focus on assessing suicidal symptoms, observing and securing the environment around the person, while the patients themselves emphasise the importance of being understood regarding what it is like to live with and deal with suicidal ideation (Berglund, Åström, and Lindgren 2016).

The use of assessment instruments for suicide risks has been described as helpful in building trust, facilitating open communication and collection of suicide‐related information (Vandewalle et al. 2021; Wärdig et al. 2022). Furthermore, they seem to increase nurses' confidence in their assessments and interactions (Wärdig et al. 2022). However, the assessment instruments are also time‐consuming (Velupillai et al. 2019) and disruptive as they tend to shift focus away from the subjective aspect of the assessment, with increased risk of overlooking important signs based on gut feelings and intuition (Vandewalle et al. 2021; Wärdig et al. 2022). Research highlights the importance of adopting a more holistic approach to suicide risk assessments, where therapeutic conversations form the foundation (Fedorowicz et al. 2023), taking into account the individual's needs and protection (Surveillance Report 2016).

Nurses in emergency psychiatric care perceived that their clinical assessment covered questions raised in the assessment instruments, why there were no needs for these (Chunduri et al. 2019). However, the nursing staff considered the assessment instruments valuable for professionals with less psychiatric experience. On the other hand, healthcare professionals and patients in outpatient psychiatric care were mostly positive towards the assessment instruments and found them useful and easy to use (Lang et al. 2009). The study further showed that the assessment instruments led to productive conversations or deeper exploration of topics that would not have been raised otherwise. There was a fear of identifying and documenting an increased risk of suicide and that the patient subsequently died by suicide. Even though asking about suicidal thoughts does not trigger suicidal behaviour (Dazzi et al. 2014), this was a fear of healthcare professionals using assessment instruments (Lang et al. 2009). In conclusion, it is evident that both assessment instruments and the establishment of interpersonal relationships play pivotal roles in the realm of suicide prevention. While assessment instruments have not been devoid of criticism, they continue to hold significant clinical utility. There remains a pressing need for deeper insights and enhanced comprehension regarding the utilisation of these assessment instruments by nursing staff within the specialised psychiatric context, particularly in their interactions with patients presenting suicidal ideation. Thus, the aim of the study was to describe nursing staffs' experiences of working with suicide risk assessment instruments.

## 3 Methods

### 3.1 Design

A descriptive qualitative method was chosen as it allows the respondents to illuminate their experiences of a phenomenon (Polit and Beck 2021). Content analysis focuses on extracting contextual information from the text and capturing its meaning. This method is useful when the aim is to describe a phenomenon in an area where existing research is limited (Hsieh and Shannon 2005).

### 3.2 Sample and Setting

In Sweden, all healthcare personnel, regardless of profession and level of care, should be able to perform suicide risk assessments (National Board of Health and Welfare 2023). These assessments should be conducted through conversations with the patient and the use of suicide risk assessment instruments. The specific instruments to be used vary between regions. Since 2017, the importance of the conversation itself has been emphasised, following a review by the Swedish Agency for Health Technology Assessment and Assessment of Social Services (Runeson et al. 2017), which highlighted the weaknesses of various suicide risk assessment instruments. Specialist psychiatry holds the treatment responsibility for suicidality, but all healthcare providers are expected to be capable of conducting suicide risk assessments.

The sample was arrived at by using the convenience sampling method. The inclusion criterion for the study was a minimum of 2 years of experience working in specialised psychiatric care. Nine participants were included in the study, comprising seven women and two men. One participant declined participation after initially responding to the invitation and expressing interest in participation. The participants had worked in specialised psychiatric care for a period ranging from three to 40 years. Three of the participants had a nursing education, while six were assistant nurses with a shorter psychiatric education, corresponding to a secondary education. These professions were involved in suicide risk assessments and had access to the same instruments for conducting these assessments. The assessments performed by these professionals took place in both inpatient and outpatient settings. Among the included participants, four worked in psychiatric inpatient care, three in psychiatric emergency departments and two in psychiatric outpatient care.

### 3.3 Data Collection

The data collection involved nine individual semi‐structured interviews conducted at two hospitals in central Sweden. Initially, letters were sent to the respective heads of the departments at both hospitals, who approved the study and then granted permission to conduct interviews within the clinics. Information about the study was posted on the region's intranet, where potential participants could express their interest in participating in the study. Those interested in participating were asked to contact the authors via email. In total, seven participants were included through convenience sampling, and two were recruited through snowball sampling, that is, asking the informants if they knew anyone who could participate in the study and who met the criterion.

The interview guide was developed collaboratively by the researchers involved in the study. The interview guide consisted of open‐ended main questions and follow‐up questions. Examples of questions included: ‘Can you tell me about your experiences of working with suicide risk assessment instruments?’ and ‘What knowledge is needed to be able to use these instruments?’ The purpose of the interview structure was to initially adopt a conversational style, allowing the interviewees to speak naturally and freely about their experiences of working with suicide risk assessment instruments. The interview structure was designed to capture the experiences of nursing personnel and their reflections on their experiences. Allowing for reflective opinions enables new knowledge to be conveyed (Patton 2015). The first two interviews were conducted as pilot interviews. Since no changes were made to the interview guide, these interviews were included in the analysis. Written informed consent was obtained before the interviews. The interviews were conducted individually by I.W and A.M.N, between October 2022 and January 2023. They lasted between 28 and 49 min and were digitally recorded. The interviews took place at the informants' workplaces.

### 3.4 Data Analysis

The data were analysed using conventional content analysis with an inductive approach (Hsieh and Shannon 2005). Conventional content analysis allows the authors to delve into the text and gain a new and increased understanding and enables the creation of categories and subcategories from the collected text data. The collected data were transcribed and read multiple times by the authors separately. The authors then discussed what emerged in the data that was relevant to the purpose of the study. The relevant parts were underlined to identify key elements that were important for the purpose. These key elements were discussed and grouped into categories, which were further organised into subcategories based on the similarities and differences. Throughout the process, the authors engaged in continuous dialogue with each other about the texts and the meaning of the units, as well as how they were related to each other, in order to discover a new whole. The completed analysis was evaluated by two co‐authors (R.W & S.H) with extensive experience in content analysis to confirm its relevance.

### 3.5 Ethics

The study has ethical approval from the Regional Ethical Review Board in Linköping, Sweden, reference number 2016/343‐31. The ethical guidelines outlined in the World Medical Association's Declaration of Helsinki (2013) were followed. Prior to the interviews, participants were provided with information stating that their participation was voluntary and that they could withdraw from the study at any time without providing an explanation. All participants signed written informed consent. Collected data were coded and securely stored with password protection, accessible only to the authors of the study. The COREQ (COnsolidated criteria for REporting Qualitative research) Checklist is a set of criteria that should be included in reports of qualitative research. The checklist was used by the authors to ensure that the study's method and analysis contained sufficient detail to achieve good reporting quality (Tong, Sainsbury, and Craig 2007).

## 4 Results

**FIGURE 1:**
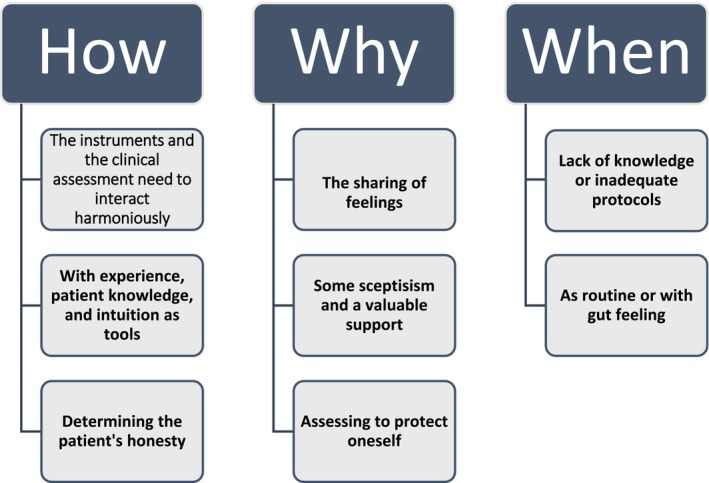
Categories and their associated subcategories.

Nine interviews were conducted with nursing staff working in specialist psychiatry, including six mental health nurses and three registered nurses. Two of the informants were male, and the remaining were females. Their ages ranged from 21 to 58 years, and they had worked in specialist psychiatry for between three and 40 years. The analysis resulted in three categories that describe how they experienced working with suicide risk assessment instruments: the how, the why and the when. The categories and their respective subcategories are presented below (Figure 1).

### 4.1 The How

This category pertains to how nursing staff experience conducting suicide risk assessments. In the assessment process, instruments serve as tools that facilitate the process, but intuition, experience and patient knowledge are also required. The assessment could also be complicated by the difficulty in determining whether or not the patient was being truthful.

#### 4.1.1 The Instruments and the Clinical Assessment Need to Interact Harmoniously

Many informants perceived the suicide risk assessment instruments as providing a solid framework to include all the necessary elements for making a well‐founded assessment. One nurse expressed complete confidence in the instruments but would combine multiple instruments when uncertain, until sufficient information was obtained to make a confident assessment. Nursing staff who used the instruments more extensively were clearer in acknowledging the need to supplement their assessments with clinical judgement.

No, I have to supplement it with my experience, absolutely. I can't just rely on the instrument. Absolutely not, never. Never actually. Because you have to have a deeper communication. (Mental health nurse, 7)

Experienced nursing staff perceived suicide risk assessment instruments as valuable tools to consider but emphasised their preference for engaging in dynamic conversations. Nursing staff from inpatient units also highlighted the opportunity they had to observe patients over a longer period, which allowed them a better chance to detect changes in behaviour and well‐being.

They talk less, withdraw more, you can see that they become more … they start isolating themselves. (Nurse, 2)

One informant experienced that the visible use of suicide risk assessment instruments could diminish trust in the clinician and create a perception of less genuine engagement. Therefore, this informant chose to always have a live conversation with patients based on the suicide risk assessment instrument and complete the rating after the patient had left.

I believe they perceive me as being incompetent and lacking genuine concern, but rather focused on simply asking questions in order to check off each step. (Mental health nurse, 5)

#### 4.1.2 With Experience, Patient Knowledge and Intuition as Tools

Experience and patient knowledge were highlighted by all informants as important factors for making a good assessment. Although suicide risk assessment instruments can be helpful, the informants experienced the importance of not becoming overly fixated on the results and the need to also incorporate their professional judgement. Despite several advantages of using suicide risk assessment instruments being pointed out, most informants believed that a natural conversation is preferable as it demonstrates comfort in discussing the subject and that it is no more awkward than asking about any other topic. Experience also meant not only being willing to ask about suicidal thoughts but also being comfortable with the answers.

Indeed, it is possible that as a patient, if the person asking the questions appears uncomfortable, one might feel inclined to protect them and, in a way, reassure them that everything is fine rather than telling the truth. (Mental health nurse, 1)

Nursing staff felt less confident in the assessment of unfamiliar patients. Furthermore, nursing staff in both outpatient and inpatient care shared that it is significantly easier to recognise suicidal signs in a patient they have met before and had the opportunity to get to know. Continuity and personal knowledge are therefore of great value. By having the nursing staff who knows the patient best conducting the assessment, deviations from habitual behaviour can be observed.

When we have had previous encounters with a patient, we can also make comparisons, such as noting if the current situation appears more severe than the previous one. This allows me to observe additional signs or changes in behaviour. For example, I might recall that during the last conversation about suicide, the patient maintained strong eye contact, but this time, the patient refuses to make eye contact. (Nurse, 8)

Having experience in psychiatric care provided a sense of confidence in knowing what to ask, how to ask the questions and what common signs to look for in assessing suicide risk. With increased experience, a gut feeling or intuition was described as being developed. This was described as a sense that something is not right, leading to concern for the patient. Such gut feelings often prompted further discussions with colleagues to express their own concerns and seek assistance in the assessment. However, when the topic of gut feeling arose, the informants were careful to emphasise that it is not a standalone assessment that can be made solely based on a feeling. One nurse shared that she tried to avoid relying solely on her gut feeling.

…One tries to rely as little as possible on gut feelings and as much as possible on factual information and medical history. If one were to solely rely on gut feelings, it would not be effective, and if one allows fear to guide the assessment, it can also compromise the assessment… (Mental health nurse 5)

On the other hand, several informants mentioned that one cannot ignore a gut feeling that something is wrong. While it is important to rely on objective information and assessments, there is recognition that intuition or gut feelings can sometimes provide valuable insights and should not be dismissed entirely.

…Absolutely, if I have a gut feeling that something is wrong and there is a potential risk for suicide, it is crucial to follow that instinct. In such cases, it is important to assess the situation as potentially risky, and signal this concern to colleagues or other appropriate healthcare professionals. (Mental health nurse, 3)

#### 4.1.3 Determining the Patient's Honesty

The informants described that, in many cases, it is difficult to assess whether or not a patient is genuinely responding honestly to questions regarding suicidal thoughts. Reasons provided include patients covering their symptoms to avoid hospitalisation, as well as exaggerating their symptoms to be admitted to the ward. When nursing staff in inpatient care were questioned, many believed that patients already admitted to the ward tend to exaggerate their symptoms in order to prolong their stay there, while conversely, some patients cover their symptoms to be discharged sooner.

Because some of our patients have the Beck Depression Inventory posted on their refrigerator at home, they know exactly what to answer to obtain the highest possible score… You can tell by the amount of time it takes to complete the BDI. Some may do it in two minutes, and then one can conclude that the patient hasn't even read the questions; they have simply marked the highest score. (Nurse, 9)

Assessing suicide risk in individuals with Borderline Personality Disorder (BPD) was perceived as an especially challenging task in light of the aforementioned observations. Some of the participants experienced that a suicidal threat expressed by a patient with BPD, in many cases, could be an expression of an entirely different need, and the task of the nursing staff becomes trying to determine what that need is, rather than providing the usual care for an acutely suicidal patient. At the same time, these patients must not be neglected regarding suicide risk, making it a balancing act.

…In patients with personality disorders, it indeed becomes crucial to identify protective factors. Often, there is a specific underlying cause behind their distress, so efforts are made to identify the triggering factor that is contributing to their current poor well‐being. (Nurse, 2)

To strengthen their suspicions that the patient is being dishonest or exaggerating, the informants described that they delve deeper into their questioning by, for example, asking about the patient's thoughts and plans for their life in the near future and beyond.

Many times, I can ask the patient if they have suicidal thoughts or plans, and follow up by asking if they have written a farewell letter or what plans they have for the upcoming month or two. (Mental health nurse, 3)

### 4.2 The Why

There were several reasons for using suicide risk assessment instruments. It was perceived, among other things, as a support for patients who struggle to articulate their emotions. It was also considered a valuable tool for inexperienced nursing staff and, importantly, a way for the informants to document that they have fulfilled their duties, although this aspect evoked mixed feelings.

#### 4.2.1 The Sharing of Feelings

The informants described that patients appreciate being able to talk about their dark thoughts, especially if they haven't had the opportunity to do so before. It was seen as a relief for them to be able to share with someone else.

These are questions that aren't comfortable for everyone to talk about. It can feel like a weight is lifted when everything is out in the open. (Nurse, 2)

On the other hand, some patients perceive it as a taboo subject and feel ashamed to admit having thoughts of ending their life. The informants shared that some patients appreciate the use of suicide risk assessment instruments because they don't have to put their difficult emotions into words in the same way as in a regular conversation. By conducting a conversation based on the instrument, these assessments sometimes became a combination of the instrument and therapeutic dialogue. It was described that some patients find it challenging to express themselves verbally and, therefore, prefer to respond in writing.

Sometimes patients have difficulty verbalizing their emotions. In such cases, using paper and pen can be the easiest way to reveal feelings. (Mental health nurse, 4)

The instruments were also seen as useful when patients avoid disclosing how bad they truly feel. When the suicide risk assessment instrument then indicates high scores, it could help the nursing staff to become aware of the severity of the patient's condition.

#### 4.2.2 Some Scepticism and a Valuable Support

The informants expressed a lack of knowledge regarding the validity of the suicide risk assessment instruments. There was also a clear distinction between nursing staff working in more independent settings, such as outpatient clinics, and those working in inpatient care. Those with more experience in using various instruments, and who did so proactively (not just upon the directive of the attending physician), generally displayed more scepticism regarding the validity of the instruments.

Many individuals are indeed afraid of conducting a suicide risk assessment due to factors like personal empathy and past experiences. However, there is a significant difference in how professionals work, particularly between those in acute psychiatric care who deal with these situations daily and those in inpatient wards. The professionals in acute psychiatric care tend to have more confidence and expertise in this area compared to staff working on inpatient units. (Mental health nurse, 1)

The suicide risk assessment instruments were primarily described as a valuable support for inexperienced nursing staff, such as newly hired individuals without prior experience in specialised psychiatric care, as well as students and trainees. This is because the instructions of the assessment instrument are often easy to follow, and they provide guidance on which questions should be asked.

…If we consider the suicide continuum, which forms the foundation of what we use, I can see why it might be perceived as somewhat for dummies'. It is straightforward, so someone who is new can simply follow the steps. Therefore, it serves as a clear instrument when provided with proper instructions. (Mental health nurse, 6)

#### 4.2.3 Assessing to Protect Oneself

Many of the informants described a sense of personal security when using suicide risk assessment instruments. It was seen as a reassurance for the nursing staff to be able to provide evidence in the form of documentation that a suicide risk assessment has been conducted, and thus they have fulfilled their responsibilities.

…If a patient still attempts suicide, despite the use of suicide risk assessment instruments, the nurse can at least say that they utilized the instrument and the patient responded in a particular manner. Even though the patient's actions were contrary to their response, the fact that the questions were asked is seen as a positive aspect. (Mental health nurse, 3)

On the other hand, it emerged that there was a perception that they had to protect themselves by using the instruments. This had gone too far and led to the focus being shifted from what was really important in the conversations, what truly matters. The informants described that it has become more important to document and prove that they had done the right thing, rather than actually helping and making a difference for the patient. Several of the informants described the necessity to do everything possible to cover their own backs and ensure they are not held responsible if a patient dies by suicide.

…Some actions must be taken because they are legally required, but they should still be carried out based on the best interests of the patient rather than our own. It is reasonable to rely on the fact that we are sufficiently skilled in our professions, so that we do not need to act solely to protect ourselves. (Mental health nurse, 6)

Furthermore, several informants highlighted the difficulties in assessing suicide risk among individuals with borderline personality syndrome (BPS). One of them explained that documenting suicide risk assessment becomes more demanding in this patient group because what the patient says is not always consistent with the assessment being made. Additionally, one nurse described it as a challenge since what the suicide assessment indicates and what the patient says can be different things, taking into account gut feeling and personal knowledge about the patient's challenges.

…Even though I have assessed the risk as low… In the event that the patient were to engage in any harmful behaviour. It becomes necessary to carefully choose my words to protect myself in writing. (Nurse, 8)

### 4.3 The When

The nursing staff described limited knowledge regarding available suicide risk assessment instruments and when to use them. They also mentioned that suicide risk assessments were sometimes conducted routinely but felt that they should be used more frequently and in different contexts of the care. The importance of being attuned to when the need arises was highlighted.

#### 4.3.1 Lack of Knowledge or Inadequate Protocols

The informants described relatively infrequent use of suicide risk assessment instruments. Among the informants, only one, working in outpatient care, routinely used suicide risk assessment instruments. Nursing staff in inpatient care stated that they did not use suicide risk assessment instruments because there was no specified routine for their use, other than the routine practice of having patients complete a suicide risk assessment upon admission. Most respondents admitted to being unaware of the available suicide risk assessment instruments and expressed uncertainty about whether instruments were considered valid. Several mentioned that they only conducted assessments when requested by a physician, which limited their reflection on when the need for assessment may arise.

… Suicide risk assessments are always conducted when patients are admitted, but additional assessments may be done if deemed necessary by the physicians. Nursing staff do not make independent decisions regarding the assessments. (Mental health nurse, 1)

#### 4.3.2 As Routine or With Gut Feeling

Both outpatient and inpatient nursing staff described that their routines involved conducting suicide risk assessment instruments during initial visits or admissions. It was also emphasised that nursing staff must be aware of the patient's condition. One informant mentioned that sometimes they have to adjust the content of a planned visit if they notice that the patient feels mentally unwell.

Indeed, in such situations where a patient comes in for a specific purpose, like relapse prevention, it is important for the staff to be flexible and responsive to the patient's emotional state. If I notice that the patient is feeling low or distressed, it becomes necessary to deviate from the usual manual‐based approach and focus on discussing the patient's well‐being instead. (Mental health nurse, 3)

Several of the informants agreed that conducting a suicide risk assessment is always valuable. One nurse described that it is better to ask one extra time as it is not possible to know what's going on in someone else's mind. Informants working in inpatient care often engaged in relaxed conversations about suicidal thoughts with their patients during daily supportive sessions, especially if the patient sought treatment for suicidal ideation. Such conversations were described as beneficially taking place while watching television together or going for a walk, as it created a more relaxed environment for the patient to speak openly.

You can take the opportunity to sneak in some questions about life and how one views it when doing something else, something together. It often leads to good conversations when you're out walking. (Mental health nurse, 5)

## 5 Discussion

The aim of the study was addressed through three main categories: the ‘How’ describes the procedures employed by nursing staff when conducting a suicide risk assessment and the methods they employ to utilise suicide risk assessment instruments; the ‘Why’ presents the experiences and justifications for the use of these instruments; and the ‘When’ explains the experiences relating to when such assessments are carried out.

In the results of our study some nursing staff described that patients may have problems articulating suicidal ideation and that suicide risk assessment instruments can be helpful for patients to begin talking about their emotions. However, the nursing staff stated that these assessments should not be used solely but with genuine engagement in the patient's life story. Communication is fundamental in psychiatric nursing and involves tailoring communication to the patient's needs and employing appropriate techniques (Travelbee 1971). Communication is described as a reciprocal process where nurses, drawing on their creativity, can guide the conversation. Earlier studies have found that patients with suicidal ideation may have difficulties expressing their thoughts. This may be due to stigma in having a psychiatric diagnosis, as well as from stigmatising feelings surrounding suicidal ideation (Carpiniello and Pinna 2017). To overcome stigma, it is important that healthcare professionals pay attention to the patient's life story and show a genuine engagement relating to what it is like to live with mental illness and suicidal ideation. This can open up roads towards recovery and coping (Tekin and Outram 2018). To do this, nursing staff must rely on the memoirs of the patients. However, determining a patient's honesty was highlighted as a major challenge, which can also possibly be seen as a form of stigma, where the patient is not trusted.

Specific difficulties were mentioned regarding the assessment of suicide risks in persons with BPS. Maintaining confidence in a low suicide risk assessment, despite the patient expressing suicidal plans, requires significant professional experience and sensitivity. In this, it is of utmost importance to know the excess risk that comes with BPS. Nearly 10% of persons with BPS die by suicide and make an average of three serious attempts during their lifetime (Paris 2019). One‐third of this group of patients shows significantly higher co‐morbidity in the form of self‐harm and suicidal ideation (Brager‐Larsen, Zeiner, and Mehlum 2023). Suicidal ideation can be identified from high emotional reactivity among patients with BPS, indicated by their difficulties in expressing positive emotions or through their depressive symptoms (Polanco‐Roman et al. 2018). Patients with BPS therefore need support to express positive emotions and improve emotional regulations to reduce the risk of suicidal ideation (Polanco‐Roman et al. 2018; Brager‐Larsen, Zeiner, and Mehlum 2023).

Nursing staff in our study also expressed concern that patients may cover their genuine level of suicidal ideation. Nursing staff emphasised the advantage of being able to observe patients over an extended period, enabling them to detect subtle changes indicative of deteriorating mental well‐being and an increased suicide risk. According to Rytterström et al. (2019), non‐verbal communication can help nurses recognise suicide plans, even when patients do not express them verbally. If the patient has trouble expressing themselves, and the nurse is unable to derive meaning from the patient's communication or does not listen sufficiently, this constitutes a nursing issue (Travelbee 1971). In such instances, the nurse must employ alternative strategies to address the patient's concerns, such as sharing perceptions or utilising open‐ended comments and questions. Donner and Wiklund Gustin (2021) argue that non‐verbal and verbal communication occur in concert and that non‐verbal cues, such as tone of voice, can reinforce or contradict verbal expressions. Non‐verbal communication can complement incomplete verbal communication or contribute to the interpretation of verbal messages. In this there is an inherent problem; for the non‐verbal communication to be counted as a finding in line with our other methods of assessment, it is necessary to acknowledge that intuition, gut feeling and experience from previous patient encounters are equally important. Previous research has described this intuition in the context of suicidal ideation (Rytterström et al. 2020), but it is primarily the suicide risk assessment instruments that are in clinical use in many different versions (Ryan and Oquendo 2020). In Sweden, the suicide risk assessment instrument plays a significant role in the overall evaluation (National Board of Health and Welfare 2023). However, in the UK, its use has been increasingly questioned and discouraged. Instead, there is a clearer emphasis on including the perspectives of the patient, their relatives and friends, due to the instrument's low validity (Fedorowicz et al. 2023). The use of suicide risk assessment instruments and nursing staffs' possible gut feelings can be complemented by thoughts about how to care for people with a ‘wish to hasten death’ due to somatic diseases (Rodríguez‐Prat, et al. 2017). A wish to hasten death can have different meanings for different people and can denote something other than being purely an expression of a death wish. Therefore, it is of utmost importance that the nurse understands the causes and reasons for this desire to die and thereby be able to develop and implement care plans that meet the patient's individual needs.

Thus, suicide risk assessment instruments and the patient's memoirs, along with nursing staff intuition, play important roles in suicide prevention work. However, it is also important that healthcare professionals are aware of their attitudes towards suicide risk assessment instruments, as these attitudes also influence clinical practice (Dickens et al. 2023). Suicide risk assessment instruments may have their place in some cases, but the focus should not be diverted from subjective assessment, where professional experience, patient familiarity, intuition or gut feelings are equally vital components of the overall assessment (Wärdig et al. 2022; Vandewalle et al. 2021). It is also important to be aware that the interpretation of the instrument assessment is likely to be influenced by the individual conducting it (Gale et al. 2016).

The results of this study show that nursing staff describe their knowledge of suicide risk assessment instruments as low, and inpatient care professionals were particularly hesitant because they were unaware of the available instruments and only conducted assessments when instructed by the responsible physician. This uncertainty regarding how, why and when suicide risk assessment instruments should be used could be seen as logical in light of the fact that the WHO (2023) has comprehensive guidelines but has not specified how these assessments should take place. The Swedish guidelines describe that suicide risk assessment instruments should be used together with the patient's story in their own words (National Board of Health and Welfare 2023). These guidelines further state that suicide risk assessments are to be documented in the patient's journal. The informants described that it has become more important to document and prove that they have done the right thing, rather than actually helping the patient. It can be considered a dangerous phenomenon if nurses feel that time must be spent on perfect documentation rather than time together with the patient. As a nurse, it is crucial to spend a significant amount of time with the patient for various reasons, even though administrative tasks are increasingly consuming a larger portion of the nurse's time (Olivares Bøgeskov and Grimshaw‐Aagaard 2019). Previous research has also shown that the documentation becomes a means for the healthcare professional to protect themself, rather than prioritise the patient's needs (Chunduri et al. 2019). A common theme in our study is also found in Lang et al. (2009), who discusses the concern among healthcare professionals about assuming responsibility if something were to happen. This could be a reason why nursing staff in this study feel compelled to use the instruments, even though they do not have much confidence in them.

### 5.1 Limitations

The inclusion in the study of participants from two different hospitals in Central Sweden can be regarded as a limitation as it does not minimise the risk of capturing a specific healthcare culture within a specific region (Patton 2015). However, the nursing staff's patient knowledge and experience may provide insights into a broader population of nursing personnel in specialist psychiatric care. One strength of the study lies in the selection of participants based on their suitability and the valuable experiences they could contribute to the research (Patton 2015). While the healthcare professionals’ experiences may have been influenced by the study's purpose, it is worth noting that convenience sampling is considered a time‐efficient and suitable method within a specific research domain, facilitating access to informants within the target population (Polit and Beck 2021). On the other hand, a purposeful sampling method might have contributed to a greater variation in participants' experiences.

To ensure methodological rigour and trustworthy results, principles of credibility and transferability were followed (Maher et al. 2018). The credibility of the study was based on how well the participants addressed the research purpose. In the concluding interviews, no new data emerged in any of the domains, indicating data saturation (Polit and Beck 2021). Irrelevant data were excluded during the analysis, while relevant data were included, ensuring the quality of the interviews (Hsieh and Shannon 2005). Including quotes from the interviews in the study's results also enhances credibility for the various categories and subcategories (Patton 2015). The quotes also strengthen the authenticity of the study, as the participants describe their experiences in their own words (Polit and Beck 2021).

Through our descriptions of the demographic background and context, readers can assess the similarity between the study's results and the potential transferability to other contexts (Patton 2015). Lastly, the selection of participants with varying ages and professional experience can contribute to different perspectives and a broader variation of the studied phenomenon.

## 6 Conclusion

Within specialist psychiatric care, the use of suicide risk assessment instruments is limited, and when used, they are always combined with the nursing staff clinical assessment. In this study, the nursing staff believed that their own clinical assessment and the natural conversations with the patients were more important than the results of the suicide risk assessment instruments. Even though the nursing staff did not have full confidence in these instruments, the Swedish guidelines require their use. In summary, suicide risk assessment instrument can be significant and helpful in certain cases, but the essential components of a suicide risk assessment consist of a conversation to understand the meaning of suicide from the patient's perspective.

## 7 Relevance to Clinical Practice

This study provides insights about how, why and when nursing staff use suicide risk assessment instruments and the interaction of these with their clinical assessments. As suicide risk assessments are one of the most complex tasks in psychiatric care, as well as in other healthcare settings, the findings can contribute to deepening the understanding of how nursing staff utilise these assessments. Suicide is irreversible, and both research and clinical practice must strive to provide the best possible care.

## Author Contributions

The authors have confirmed that all authors meet the ICMJE criteria for authorship credit (www.icmje.org) as follows: (1) substantial contributions to the conception and design of or acquisition of data or analysis and interpretation of data, (2) drafting the article or revising it critically for important intellectual content and (3) final approval of the version to be published. The study was designed by all authors. Data were collected by Isabella Wallerstedt and Anna Mattison Nyström and analysed by all authors. All authors drafted the manuscript. All authors interpreted the results, reviewed and commented on multiple versions of the manuscript and approved the final version.

## Conflicts of Interest

The authors declare no conflicts of interest.

## Data Availability

The data that support the findings of this study are available on request from the corresponding author. The data are not publicly available due to privacy or ethical restrictions.
